# A shared vision for macromolecular crystallography over the next five years

**DOI:** 10.1063/1.5131017

**Published:** 2019-12-04

**Authors:** Andreas Förster, Clemens Schulze-Briese

**Affiliations:** DECTRIS Ltd., Täfernweg 1, 5405 Baden-Dättwil, Switzerland

## Abstract

Macromolecular crystallography (MX) is the dominant means of determining the three-dimensional structures of biological macromolecules, but the method has reached a critical juncture. New diffraction-limited storage rings and upgrades to the existing sources will provide beamlines with higher flux and brilliance, and even the largest detectors can collect at rates of several hundred hertz. Electron cryomicroscopy is successfully competing for structural biologists' most exciting projects. As a result, formerly scarce beam time is becoming increasingly abundant, and beamlines must innovate to attract users and ensure continued funding. Here, we will show how data collection has changed over the preceding five years and how alternative methods have emerged. We then explore how MX at synchrotrons might develop over the next five years. We predict that, despite the continued dominance of rotation crystallography, applications previously considered niche or experimental, such as serial crystallography, pink-beam crystallography, and crystallography at energies above 25 keV and below 5 keV, will rise in prominence as beamlines specialize to offer users the best value. Most of these emerging methods will require new hardware and software. With these advances, MX will more efficiently provide the high-resolution structures needed for drug development. MX will also be able to address a broader range of questions than before and contribute to a deeper understanding of biological processes in the context of integrative structural biology.

## INTRODUCTION

Macromolecular crystallography (MX) has been singularly successful in letting scientists determine the three-dimensional structures of biological macromolecules (proteins, DNA, and their complexes) at resolutions that allow the placement of individual atoms. The resulting atomic structures reveal the chemical basis of the enzyme function, help explain the functioning of molecular machines, illuminate the molecular basis of dysfunction in diseases, and are used for the development of vaccines and drugs. They have generally furthered our understanding of biology quite dramatically.

For most of the history of structural biology, MX has stood supreme. It was the method that achieved the highest-resolution information and gave the most reliable structures, while not suffering from any fundamental limitation on sample size or nature—as long as the sample could be crystallized. Alternative methods such as nuclear magnetic resonance spectroscopy, small-angle X-ray scattering, electron cryomicroscopy (cryo-EM), and mass spectrometry were considered supplementary or niche.

Over the last few decades, most data collection for MX has been done at synchrotron beamlines. These resources have seen impressive technical improvements over the years. They provide users with X-ray beams of highly desirable properties, such as high photon flux, low divergence, a high degree of stability, adjustable energy, and beam diameter adjustable down to a few micrometers. Coupled with highly experienced beamline staff, powerful automation and remote control systems, fast detectors, and expert processing pipelines, progress at synchrotron beamlines has removed most technical obstacles to MX. Scientists do not go to the synchrotron to do MX, but to obtain structures.

For the most part of this development, access to beamlines has been limiting. Users would need to compete for beam time based on the scientific merit of their projects. While large institutes would often make a shared case for access and gain regular access that would be spread among their member laboratories, small individual laboratories would sometimes have to wait for months to access a beamline.

Despite its maturity and success in answering biological questions, MX has now arrived at a critical juncture. Three main developments are changing the context in which MX is being done. First, cryo-EM has made dramatic advances over the last five years and is now, as a method, at least equal to MX for the purpose of determining the structures of the most interesting biological complexes. Second, synchrotrons worldwide are undergoing upgrades that will increase the photon flux of their beamlines and thus decrease the time it takes to collect data. Third, X-ray free-electron lasers (XFELs) have changed the way structural biologists think about sample and data collection. As a result of these three developments, beam time is expected to grow faster than the user demand for it, and beamlines must innovate and broaden their scope or specialize to provide the most valuable service to their users.

In this paper, we will discuss how MX might evolve over the next five years. To do this, we will start by looking back five years and summarizing the points that have taken MX where it is now and that challenge its primacy. We will then give a brief overview of the current state of MX, with a particular focus on collection, processing, and archiving of diffraction data. With the scene thus set, we will outline the ways in which MX might transform over the next five years. This speculative section combines our vision with community contributions gathered in discussions, talks, and other contributions.

In the future, we expect traditional rotation crystallography to remain the most widely used method for solving protein structures, but formerly niche applications will rise in prominence as beamlines specialize. We expect serial crystallography, pink-beam crystallography, and crystallography at energies above 25 keV and below 5 keV to become routine at dedicated beamlines and make experiments routinely possible which are currently only carried out as demonstration studies. Naturally, most of these emerging methods will require new hardware and dedicated or improved software solutions, but there is nothing fundamentally hypothetical about them. They present a clear way forward for MX, which will continue to thrive not only as one aspect of integrated structural biology but also as a powerful method by itself. Scientists of all kinds will benefit from more and better data with which to answer biological questions.

## MX IN THE PAST FIVE YEARS

To develop a vision for MX for the next five years, it is worthwhile looking back the same amount of time. In 2014, buoyed by innovation, MX occupied a position of primacy among structural biology techniques. Scientists successfully worked with more-marginal samples (smaller crystals and larger unit cells) and obtained the highest-resolution structures from ever more complex samples (e.g., Pflug *et al.*, 2014; Robart *et al.*, 2014). Other established structural techniques were complementary and helped bolster findings made by MX.

Chief among the technological innovations that benefited MX are arguably hybrid photon counting (HPC) detectors, such as PILATUS and EIGER (Förster *et al.*, 2019). Introduced in 2006, they changed the way data are collected and improved the data quality that can be achieved. Their short readout times allowed for shutterless data collection, which avoided errors due to shutter jitter. The lack of readout noise resolved the trade-off between the number of images and the rotation increment per image (Pflugrath, 1999) and let experimenters take full benefit of fine slicing (Casanas *et al.*, 2016; Mueller *et al.*, 2012). The data quality that could be obtained with these detectors marked a quantum leap from earlier technologies, such as CCD detectors. At the IUCr Congress in Montreal in 2014, EIGER, the current generation of HPC detectors, was presented to the crystallographic community.

At around 2014, the transition from CCDs to the more powerful HPC detectors was under way at many beamlines. The same happened on a smaller scale in academic environments, with the diffractometer manufacturers Rigaku and STOE quick to see the advantages of the new technology for laboratory users. Data quality improved, and methods that depend on the highest data quality, such as native single-wavelength anomalous dispersion (SAD), made a resurgence (Rose *et al.*, 2015).

The higher frame rates mean that HPC detectors were capable of necessitated novel ways of handling data. Instead of individual diffraction images saved as single files, EIGER saved entire datasets inside HDF5 containers. This required visualization software, processing pipelines, and users to adapt. The result of considerable effort was easier archiving of data, more comprehensive metadata, and more efficient data input/output operations.

Other technological advances enhanced beam stability and made smaller beam diameters possible. They allowed for the construction of true microfocus beamlines where crystals a few micrometers across could be studied (Perrakis *et al.*, 1999). The low total diffraction power of microcrystals drove the establishment at synchrotrons of serial crystallography (Rossmann, 2014). From 2014, the first groups were succeeding in assembling complete datasets from crystals that were individually much too small to provide them (Roedig *et al.*, 2015; Zander *et al.*, 2015). In a way this was a journey back in time as much of MX was necessarily done with multiple crystals before the advent of cryocooling, but the process had now become faster, better controlled, and more compelling because of better analysis tools.

For the reasons just outlined, MX had reached a pinnacle, but challenges were building from three unexpected sides. The first XFEL suitable for MX had come online in 2009 and started producing data good enough for the solution of protein structures over the next few years (Boutet *et al.*, 2012; Chapman *et al.*, 2011; Liu *et al.*, 2013). By 2014, it had become clear that XFELs were extraordinary tools for MX (Neutze, 2014), in particular for dynamic studies, although their low throughput with very few facilities worldwide with only a few beamlines each limited their reach.

In 2012, the first direct electron detectors using CMOS-based monolithic active pixel sensors were sold for use in cryo-EM. These detectors were the first to achieve detective quantum efficiency higher than films (McMullan *et al.*, 2014). In addition, their high frame rates made it possible to acquire data as stacks of movie frames instead of individual images as before. With these movies, beam-induced drift could be corrected (Li *et al.*, 2013). The maximum resolution obtainable from cryo-EM reconstructions jumped almost overnight from something around 10 Å to close to 3 Å. With the structure of the mammalian transient receptor potential channel TRPV1, the resolution barrier to the visualization of side chains was finally broken (Liao *et al.*, 2013). In 2015, the first cryo-EM reconstructions to better than 3 Å resolution were published (Bartesaghi *et al.*, 2015; Campbell *et al.*, 2015; Fischer *et al.*, 2015; Grant and Grigorieff, 2015; Jiang *et al.*, 2015; Yu *et al.*, 2015). Crystallographers were taking note.

The third technique that caught crystallographers' attention in 2014 was electron diffraction (ED), when the first protein structures were determined from diffraction data collected inside an electron microscope first with a tilt series of still images (Shi *et al.*, 2013) and then with the familiar continuous rotation method (Nannenga *et al.*, 2014). All three emergent techniques outdid traditional MX in one critical point: they did not require large single crystals of the macromolecule or complex of interest. The question of whether they would be complementary with MX or supersede it hung heavy over the community.

## CURRENT STATE OF MX

**FIG. 1.**
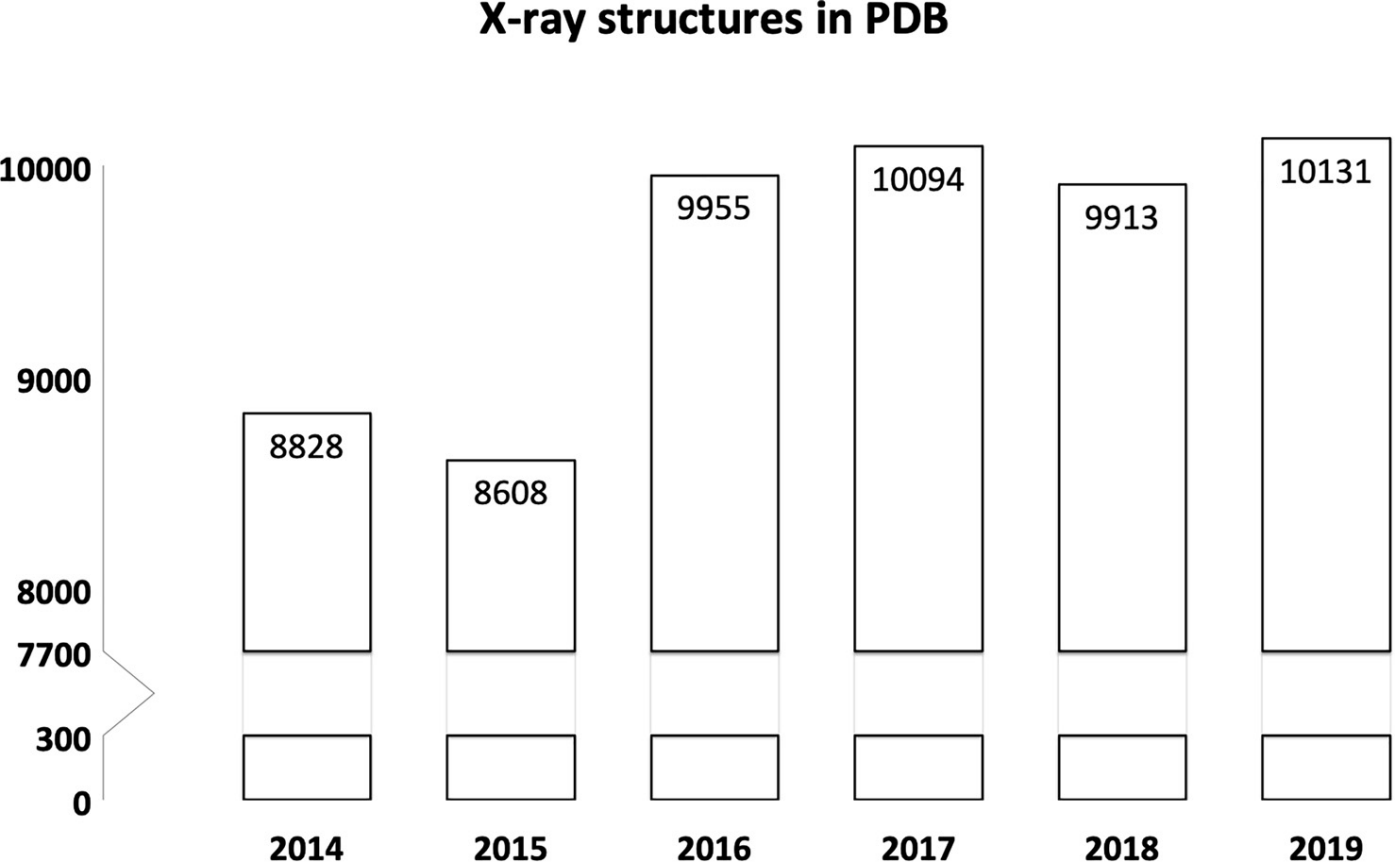
Crystallography is going strong. The number of entries released annually by the PDB since 2014 is shown. The number for 2019 was obtained by extrapolation from September 4, 2019.

As outlined in “MX in the past five years,” laboratory- and synchrotron-based MX were highly successful in 2014 but faced competition from XFELs, cryo-EM, and ED. With these three methods causing excitement, securing funding, and issuing impressive publications, it is tempting to be worried about MX. This would be wrong. MX is as strong as ever (Fig. 1), and the amount of innovation taking place around MX is not any smaller than in the three alternative methods introduced above.

It could thus be argued that there has never been a better time to do MX. The method has reached such maturity that it is frequently considered a commodity whose practice is offloaded to technicians or automated pipelines. Entire beamlines have been built around the idea that expert users are dispensable (Bowler *et al.*, 2015). This frees the scientist to think about the biological problem under study and address it with a wider arsenal of structural methods.

## VISIONS OF STRUCTURAL BIOLOGY

In the past, MX was an important method for achieving scientific breakthroughs. The first atomic structures of the ribosome (Ban *et al.*, 2000; Wimberly *et al.*, 2000), of intact viruses (Abad-Zapatero *et al.*, 1980), of antibodies bound to cognate antigens (Colman *et al.*, 1987), of channels and transporters (Abrahams *et al.*, 1994; Weiss *et al.*, 1991), of complexes involved in photosynthesis (Deisenhofer *et al.*, 1985), and of many more biologically important macromolecules were all determined by MX. In the days of blobology, there was no alternative. This has now changed.

Many of the most interesting biological systems are large, complex, and difficult to crystallize, the latter often because of the inherent mobility of the system. Now that it routinely reaches resolutions close to those obtained with MX, cryo-EM is a more obvious technique to study large molecular machines, membrane protein complexes, and cellular features. While we will continue to see high impact publications whose structural information is entirely based on MX, it is likely that structures of protein complexes of outstanding biological interest will predominantly be determined by cryo-EM.

At the same time, cryo-EM is not a solution to all problems. The bulk of solved structures is of small proteins or complexes. In the Protein Data Bank (PDB), more than 90% of all X-ray structures are of proteins or complexes smaller than 200 kD. The vast majority of EM structures, in contrast, is of complexes larger than 200 kD. This distribution is likely to persist in the future, given the smaller crystals and lower resolution obtained by MX for proteins larger than 200 kD (Svensson *et al.*, 2019). Far from displacing MX, cryo-EM is rather extending the range of samples that can be studied toward the larger, more complex, and arguably biologically more interesting end. At the same time and despite ongoing technical advances, cryo-EM is poorly suited to the solution of proteins much smaller than 100 kDa. MX will thus remain responsible for the bulk of structure deposition in the future (Shoemaker and Ando, 2018).

There are other drawbacks to cryo-EM. Cryo-EM does not work at room temperature because the illumination of single particles by electrons causes more severe radiation damage than the exposure of crystals to X-rays (Henderson, 1995). Cryo-EM cannot easily determine the identity of metal ions in enzymes. Finally, despite publication titles promising atomic-resolution structures, cryo-EM is still a medium-resolution structural method. For high-resolution information, MX is unsurpassed. For comprehensive answers to biological problems, it is thus best to combine techniques.

Cryo-EM and MX are complementary and should be used in conjunction, but they are not the only techniques to determine biological structures. The term integrated structural biology has been coined to describe an approach where information across the biological hierarchy, from atoms to organelles, is combined to understand the cell. Most interesting to crystallographers are small-angle X-ray scattering, atomic force microscopy, and electron cryotomography that often result in envelopes suitable for molecular modeling (e.g., Faelber *et al.*, 2019). This process can be expanded to include information from nuclear magnetic resonance spectroscopy, mass spectrometry, correlated light and electron microscopy (CLEM), super-resolution light microscopy, small-angle X-ray scattering, and virtually any structural, biophysical, or theoretical approach (Rout and Sali, 2019).

## SPECIALIZATION OF BEAMLINES

So far, we have made the case that recent development in MX has been rapid and that the range of problems in biology which can be addressed structurally has grown. We now want to discuss the place of MX in the bright future of structural biology. Most synchrotron beamlines are currently all-purpose experimental facilities that will accommodate their users' needs. Some beamlines offer serial crystallography, pink beam data collection, data collection at room temperature, or data collection at low energies. We think that this specialization will only increase in the future.

### Single-crystal rotational crystallography

The traditional way of collecting data by rotating the sample while it is exposed to X-rays (Arndt *et al.*, 1973) is likely to remain the mainstay for many beamlines. It is fast and easy and does not require high flux. In fact, high flux is likely to cause lower data quality, primarily because of increased radiation damage (Winter *et al.*, 2019). The vast majority of structures in the PDB is and will continue to be determined this way, although structural biologists will think twice before attempting to crystallize proteins or complexes larger than 120 kDa (Svensson *et al.*, 2019). They are now more likely to get good structures from larger molecular complexes with cryo-EM.

Despite easy access to sophisticated MX beamlines, many structural biology laboratories continue to see value in their diffractometers and keep upgrading or replacing them. Compared to synchrotron beamlines, laboratory diffractometers suffer from a larger beam diameter, greater wavelength dispersion, and a higher beam divergence and will generally produce data of slightly lower quality. On the upside, the flux of modern sources, such as a powerful rotating anode or a liquid-metal jet generator, is comparable to that of beamlines at a second-generation synchrotron, and a laboratory HPC detector represents the same state of the art as its synchrotron counterpart.

In many cases, a laboratory diffractometer can be a powerful instrument for structure determination. It cannot match the productivity of highly automated synchrotron beamlines but can do much more than optimizing crystallization and cryocooling conditions of crystals destined for data collection at a synchrotron. The three main applications of a laboratory diffractometer are data collection at room temperature, projects that require a quick turnaround, and the training of crystallographers.

Data collection from single crystals at room temperature (see below) is arguably easier in the laboratory than at a synchrotron. Because of the low flux, crystals will survive minutes or hours in the beam when they would die after milliseconds when exposed to the full flux of a modern MX beamline. A number of innovations have made it possible to reliably collect high-quality room-temperature data. Mitegen sleeves will keep crystals hydrated while being easier to work with and causing much less background than the glass capillaries of yore (Rajendran *et al.*, 2011). A more advanced solution is electronic humidity control devices such as those commercialized by Arinax and Mitegen (Russi *et al.*, 2011).

A laboratory diffractometer is indispensable for projects that require a quick turnaround, where experiments are designed based on structural information. This could be the characterization of enzyme activity with the help of mutations to the active site whose structural effects are studied (e.g., Lubkowski *et al.*, 2019). It could also be a drug discovery project where, based on structural information from cocrystal structures, intermediate leads are synthesized and improved on the way to a promising drug candidate (e.g., Pagare *et al.*, 2018).

The third potential benefit of a laboratory diffractometer is the training of crystallographers. In spite of increased automation (of data processing, crystal mounting, or complete beamline operation), knowledgeable and experienced crystallographers are still needed. They know how to solve problems, handle difficult projects, and find ways of taking marginal datasets to something sufficient to solve a structure and answer biological problems. Despite their commendable efforts at education (e.g., RapiData and Smith *et al.*, 2010), synchrotrons cannot provide this service. Experiments are over too quickly for students to think about and rationalize what is happening. At their home institution, they can just go to the diffractometer or consult with a more senior crystallographer whenever a question pops up. With a laboratory diffractometer, students have the time to focus on learning and understanding.

This is especially important with the increasing popularity of mail-in data collection for routine experiments. This mode of operation where samples are shipped to the synchrotron and data collection is controlled remotely offers important advantages to structural biologists. Costs associated with travel and accommodation are minimized, no time is lost traveling to the synchrotron and getting trained on site, and data are collected in a more reproducible way, especially when the experiments are performed in a fully automated way (Bowler *et al.*, 2016). All crystallographers should welcome remote-access data collection, unless they do complicated experiments, such as serial or room-temperature crystallography, which need scientists on site. The only drawback of remote access is the lack of hands-on experience for structural biologists and the loss of crystallographic expertise in the community over time. Besides crystallography courses and schools organized at synchrotrons, a well-equipped home source is critical for the training of the next generation of crystallographers.

### High-throughput screening

High-throughput ligand and fragment screening have long played an important role at MX beamlines thanks to automatic crystal mounting and centering and fast data collection. Offering services to the pharmaceutical industry is also an attractive way to connect a beamline to the world outside academia and to supplement its budget. High-throughput structure-based drug discovery started out in pharmaceutical laboratories (Nienaber *et al.*, 2000) but took off at standard MX beamlines (Kuhn *et al.*, 2002). The process has evolved over the years, with a critical step being the realization that full structural refinement is essential to the reliable identification of weakly bound ligands (Schiebel *et al.*, 2016).

**FIG. 2.**
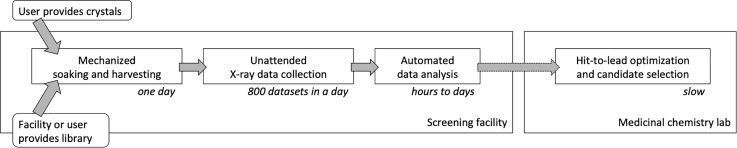
High-throughput screening workflow. Dedicated high-throughput screening facilities associated with synchrotron beamlines can accelerate hit identification (included in step “Automated data analysis”) to less than a week. Hit-to-lead optimization and candidate selection are currently still slow. This figure is based on the material provided by Frank von Delft (Diamond Light Source).

In the future, we will see that more beamlines specialize in high-throughput screening. They will follow the lead of beamline I04-1 at Diamond Light Source (DLS), which is an integral part of the XChem facility (Fig. 2). In XChem, every step from ligand soaking (Collins *et al.*, 2017) to data analysis has been optimized and is tracked with a database-backed graphical user interface (Krojer *et al.*, 2017). The collected data are analysis in a holistic way, with datasets of targets free of ligand giving valuable information on the structure and content of the nominally empty binding site, which can be subtracted from the dataset of hits to reveal the structure of the ligand (Pearce *et al.*, 2017).

With the hardware and software developments implemented in XChem, high-throughput screening has become unrecognizable except for one important aspect. Large, single crystals are used for data collection. There is no reason that needs to remain the case. As screening campaigns already involved hundreds of crystals, it makes sense to take advantage of serial crystallography approaches. For example, one can transfer crystals to a micromesh or other targets suitable for serial crystallography by acoustic droplet ejection (Cuttitta *et al.*, 2015), add ligands in a similar way, and collect all data from one or a few mounted meshes, avoiding the time it takes to mount and dismount all crystals individually.

Despite considerable excitement about its prospects (Boland *et al.*, 2017), it is unlikely that cryo-EM will contribute meaningfully to high-throughput screening in the near future. As long as well-diffracting crystals of the target protein exist, MX will collect hundreds or even thousands of datasets and determine structures quickly and automatically (Schiebel *et al.*, 2016). It is likely that cryo-EM complements MX during the early stages of drug discovery projects (Ceska *et al.*, 2019), but MX will remain the essential tool.

### MX at high energies

An exciting development in MX is data collection at energies above 20 keV. Based on Darwin's formula (Darwin, 1922), which encapsulates the physics of diffraction, Arndt (1984) suggested that the diffraction power of a crystal grows faster than the absorbed dose as the energy of the incident photons is increased to about 30 keV. Diffraction efficiency (DE), defined as the number of elastically scattered photons per dose, increases because photoelectric absorption (which is responsible for radiation damage) falls faster than elastic scattering (which gives rise to Bragg peaks) with increasing photon energies. At even higher energies, Compton scattering, another process causing radiation damage, becomes dominant, which leads to a decrease in diffraction efficiency. The energy to maximize DE has recently been calculated to be about 35 keV (Dickerson and Garman, 2019).

While it should permit the collection of more diffraction data from a single crystal before the onset of global radiation damage, this observation was of limited practical relevance in the past as the existing X-ray detectors had poor detection efficiency at high energies. With the appearance of cadmium telluride (CdTe)-based detectors, this has changed. Excellent MX data can now be collected at high energies (Takaba *et al.*, 2019).

**FIG. 3.**
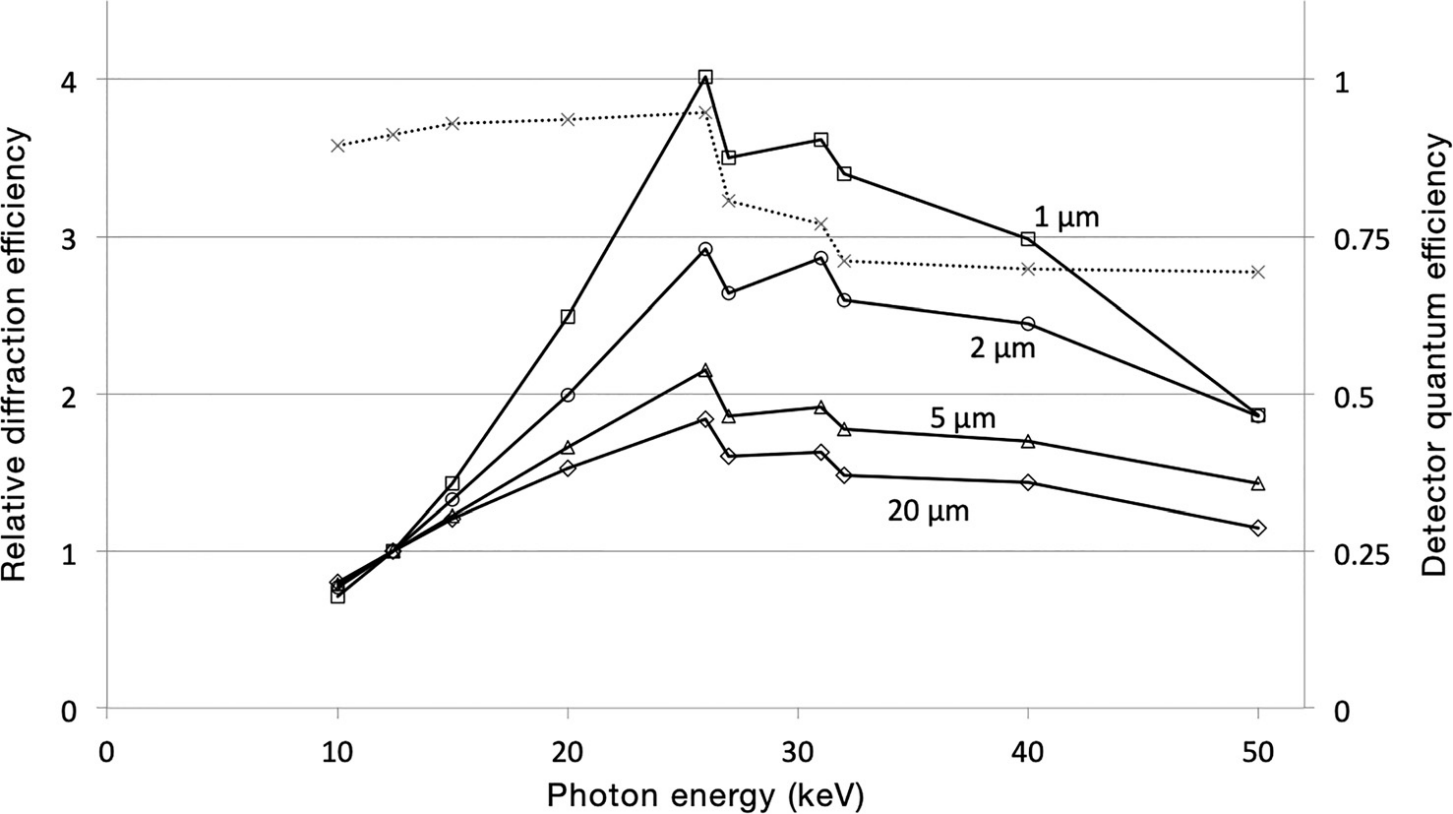
The best energy for MX. The diffraction efficiency (i.e., the number of elastically scattered photons normalized to the absorbed dose) increases with the increasing photon energy up to around 30 keV. Shown are the results of Monte Carlo simulations for crystals of various sizes (1 *μ*m, squares; 2 *μ*m, circles; 5 *μ*m, triangles; and 20 *μ*m, rhomboids). The effect is compounded for microcrystals (d ≥ 2 *μ*m) with significant photoelectron escape. The data (kindly provided by Joshua Dickerson) take the quantum efficiency of a HPC detector with 750 *μ*m thick CdTe sensors into account (dotted line, right axis) and suggest 26 keV as the best energy for MX to minimize radiation damage.

CdTe at thicknesses commonly used in hybrid photon counting detectors (750–1000 *μ*m) captures nearly 80% of photons at energies up to 80 keV and nearly all of them at energies below the absorption edge of Cd at 26.7 keV. Photons more energetic than 31.8 keV (Te edge) cause Cd and Te fluorescence, most of which is reabsorbed in the sensor material. Fluorescence thus causes some photon loss but, in particular, an unsharpening of the signal (lower modulation transfer function) that will decrease the maximally achievable data quality. This immediately suggests 26 keV as the most promising energy for MX experiments (Fig. 3), although the practical consequences of collecting data at, say, 30, 35, or 40 keV need to be determined experimentally.

The possible practical effect of collecting data at higher energies was recently simulated. The improvement in DE is expected to be small for crystals larger than about 10 *μ*m across. The effect is much more pronounced for crystals smaller than 5 *μ*m. There, photoelectron escape removes potentially highly damaging secondary electrons. This helps increase DE by a factor of up to five (Dickerson and Garman, 2019).

Other advantages of going to higher photon energies are lower absorption and lower extinction effects (Fourme *et al.*, 2012) and, thanks to Bragg's law, an increased sample-to-detector distance, which helps beamlines get more accessory equipment into their end stations without having to worry about collisions. The primary disadvantage of higher energies is a decreased anomalous signal for the vast majority of elements. Experimental phasing is expected to work only in exceptional cases with data collected at 26 keV. In addition, photon flux is often lower at higher energies, but for single crystal applications, essentially no beamlines are flux-limited these days.

During the next years, the experimental focus will probably be on assessing the practical advantages of collecting data at higher energies. We expect beamlines to optimize the high end of their spectrum and equip their end stations with CdTe detectors. Because of the crystal size-dependence of the beneficial effect of higher data collection energies, it seems tempting to combine data collection at high energies with serial crystallography on microcrystals. Currently, no beamline exists to offer this capability.

### MX at low energies

Despite the case made in the previous paragraph for collecting data at energies just below the Cd edge, beamlines may also specialize at the opposite end of the spectrum. Data collection at energies lower than 6 keV (corresponding to wavelengths higher than 2 Å) is attractive for experimental phasing by anomalous dispersion from heavy atoms native to proteins or nucleic acids (any bound metal, sulfur or phosphorous). This idea is epitomized by beamline I23 at Diamond Light Source (DLS), arguably the most highly specialized MX beamline in the world (Wagner *et al.*, 2016).

**FIG. 4.**
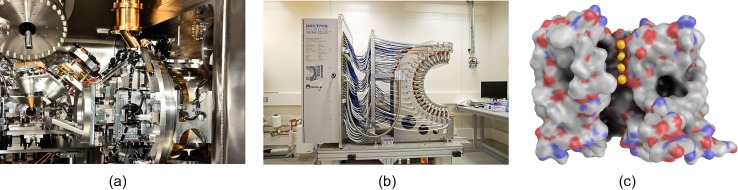
Low-energy data collection helps identify ions. (a) Beamline I23 at Diamond Light Source is optimized for data collection at energies down to 2.1 keV. A view of the goniometer shows the copper elements (golden) that are required for conductive cooling of the sample in vacuum. (b) A bespoke curved detector is used to capture high-resolution reflections at low photon energies. (c) Anomalous occupancy refinement of the four potassium ions (shown in gold) in the pore; the potassium channel NaK2K proved that they are present at one-quarter occupancy each. Single-wavelength anomalous dispersion data were collected at 3.35 Å. For clarity, one of the four protomers of the channel is not shown. Images (a) and (b) courtesy of Armin Wagner, Diamond Light Source.

The end station of I23 is entirely in vacuum to avoid air scatter and absorption. With a cryostream impossible in vacuum, the sample is cooled by conduction through copper links [Fig. 4(a)]. The beam energy is tunable between 2.1 and 12 keV. The detector is a bespoke HPC detector with a low-energy calibration and 120 modules arranged to mimic the surface of a semicylinder, so as to record the high-angle spots that even medium-resolution Bragg reflections produce at low energies [Fig. 4(b)]. The low-energy calibration ensures a reliable threshold setting and accurate data even close to the electronic noise of the readout chip (Kraft *et al.*, 2009; Marchal and Wagner, 2011).

Besides the obvious case of increasing the anomalous signal of intrinsic sulfur atoms (Aurelius *et al.*, 2017), low-energy MX can serve to identify ions [Fig. 4(c)]. This can help us answer questions as simple as how many potassium ions are inside a channel at any time (Langan *et al.*, 2018). It can also help us resolve the importance for the structure and catalytic activity of the ribosome of potassium ions that were erroneously labeled magnesium and water in earlier structures (Rozov *et al.*, 2019). In addition, weak anomalous signal might be used to assess protein side chain disorder, which has implications for the understanding of the dynamics of protein-protein complexes (Salmon *et al.*, 2018).

Beamlines that operate at energies much lower than 6 keV will grow in popularity in the future, although the technical difficulties to their success are substantial. Air needs to be removed at least between the sample and the detector, and the detector itself needs to be calibrated for low energies and be able to cover high diffraction angles. While beamline I23 at DLS serves as a prototype, simplifications to its design are possible. Beamline 1A at Photon Factory operates in a helium atmosphere and sports two stock HPC detectors that can be arranged at a 25° angle with respect to each other (Basu *et al.*, 2019). No matter the beamline specifics, low-energy MX is a decidedly low-throughput application where difficult problems with high impacts will be solved.

### Crystallography at ambient temperature

Cryocrystallography was introduced at MX beamlines more than 30 years ago to minimize the detrimental effect of X-rays on the crystals of biological materials and extend their lifetime in the beam (Hope, 1988). Data collection at 100 K has become nearly ubiquitous since then. This orthodoxy is now being questioned. On the one hand, more efficient detectors and the minimization of other errors let the experimenter get away with much less data than was required in the past and thus collect entire datasets from uncooled crystals. On the other hand, serial crystallography (see “Serial crystallography”) sidesteps radiation damage by combining the undamaged parts of datasets from many crystals.

Collecting data at room temperature has an unexpected advantage. In contrast to what is the case at cryogenic temperatures, global radiation damage and specific radiation damage are not uncoupled. If the recorded intensities are stable across the experiment, there is little specific radiation damage and thus only a few radiation-induced structural artifacts that complicate the interpretation of the results (Gotthard *et al.*, 2019). This can help, in particular, with the study of metal sites in proteins.

Cryocooling alters the structural ensembles of side chains in protein crystals (Fraser *et al.*, 2011). This poses the danger of drawing the wrong conclusions with respect to catalysis and protein-protein interactions. Data collection at room temperature makes sure we get a physiologically relevant view of the protein surface. If data are collected at a number of different temperatures, from below to above ambient, temperature-dependent structural biology can be done (Keedy, 2019). For this to work, the crystals have to be sufficiently large (dose is energy absorbed per unit mass) or sufficiently many (serial crystallography). For work on single crystals, it helps us to minimize beam intensity during data collection. In other words, top-of-the-line laboratory X-ray sources with good optics, stable beams, and readout noise-free HPC detectors are the equipment of choice to do temperature-dependent structural biology from single or few crystals.

Single-crystal data collection at ambient temperature will remain a niche application, but various serial crystallography approaches will make sure that the resurgence of room-temperature data collection is strong and persistent. Obtaining atomic structures of macromolecules at physiological temperatures is something cryo-EM by definition cannot do.

### Serial crystallography

The main problem with MX has been the need for large, single crystals. Since the advent of microfocus beamlines (Perrakis *et al.*, 1999) and highly sensitive HPC detectors, much smaller, more easily grown crystals can be studied. As the number of photons that can be scattered by a crystal depends on its size, smaller crystals yield less complete or lower-resolution datasets. As crystals decrease in size, so does the information in the dataset, until data from a single crystal are insufficient to solve the structure. The solution is to combine partial datasets from multiple crystals, with the extreme case being a single image per crystal (Stellato *et al.*, 2014).

After it was somewhat forgotten at synchrotrons when cryocooling dramatically increased the lifetimes of single crystals (Hope, 1988), serial crystallography was rediscovered at XFELs (Chapman *et al.*, 2011). XFELs are ideally suited to single-shot serial crystallography because the high instant flux of femtosecond X-ray pulses deposits into each crystal a dose much beyond what causes radiation damage. The crystals are vaporized upon exposure but not before the diffracted photons escape. Cryocooling becomes unnecessary, and the crystals essentially diffract much beyond their dose limit. There are examples where XFEL data have extended to higher resolution than comparable synchrotron data (Martin-Garcia *et al.*, 2017), but XFEL beamlines are unlikely to become facilities for routine MX. Their high cost and limited availability will probably restrict their use to time-resolved studies in the femto- to picosecond range that synchrotrons cannot reach. In addition, as diffraction occurs before radiation damage sets in, XFELs will be used to study radiation-sensitive structures, such as metal-containing active centers of enzymes. Together with the high temporal resolution of XFELs, such studies will provide an unprecedented understanding of enzymatic mechanisms.

For serial crystallography to break through at synchrotrons, the community needs to embrace microcrystals. This is not as trivial as it sounds, especially for researchers used to growing large crystals. Their equipment and workflows are optimized for large crystals. Light microscopes are poor tools for working with homogeneous showers of microcrystals. Experimental protocols need to be adjusted to help researchers identify promising microcrystals when before they screened their crystallization trials for sizeable single crystals. It is likely that crystallographic suppliers will enter the market with solutions to make working with microcrystals as routine as with large crystals.

Many technological solutions already exist for working with microcrystals once they have been grown. They can be acoustically ejected from the crystallization drop onto a sample holder mesh (Roessler *et al.*, 2013) or into precise positions of a fixed-target sample holder (Davy *et al.*, 2019), injected into the beam in a liquid or high-viscosity jet (Weierstall *et al.*, 2012), or presented on a large variety of fixed targets with predetermined or random crystal positions (Hunter *et al.*, 2014). Beamline scientists have been inspired by technologies developed at XFELs and optimized them for their beamline's needs (Gao *et al.*, 2018). This spirit of innovation will lead to various beamlines being uniquely suited to certain aspects of serial crystallography.

We expect serial crystallography to rise in prominence. Not only does it yield structural information from small and weakly diffracting crystals but it also supports the study of structural polymorphs and dynamics, and it is extremely well suited for microdiffusion experiments for the study of enzyme kinetics and dynamics (Mehrabi *et al.*, 2019). Beamlines at particularly hot synchrotrons will focus on single-shot serial crystallography at physiological temperature. Other beamlines will let the user collect tiny wedges of data from a large number of possibly cryocooled crystals. On the side of the end user, crystallographers will know how to evaluate their crystallization screens for both large, single crystals and promising microcrystals.

### Pink-beam MX

While XFELs deliver all the photons needed for diffraction within a ten- to twenty-femtosecond pulse, several microseconds of X-rays are needed at current synchrotrons to yield measurable diffraction. Using multilayer optics to create a beam with a wide energy bandwidth is one way of bridging this gap between synchrotrons and XFELs. While normal monochromatic beamlines have a bandwidth of around 10^−4^, the so-called pink beamlines have bandwidths between 0.5% and 5% and can provide up to 100 times more flux (Meents *et al.*, 2017). Thanks to the high flux, exposure times of as low as 100 ps are currently possible. These are expected to decrease further at synchrotrons with upgraded lattices.

For short-pulse pink-beam serial crystallography, as well as for crystallography at XFELs and possibly other serial crystallography applications, it is necessary to use integrating detectors (Tolstikova *et al.*, 2019). Counting detectors cannot separate individual photons at these rates. It is likely that some of the detector developments for XFELs will be commercialized for use at serial crystallography beamlines at upgraded synchrotrons.

Pink-beam crystallography suffers from a number of drawbacks. Data processing is not as straightforward as for data from a monochromatic beam, and the background is bound to be higher because all photons contribute to it, while only a narrow band enters each Bragg reflection. Nevertheless, the promise of doing serial crystallography faster and with fewer samples will keep crystallographers innovating in this field.

### Electron diffraction

Similar to serial crystallography, ED does not require large crystals and thus widens the central bottleneck of MX. The reason is not the combination of partial datasets from multiple or countless crystals but the stronger interaction between electrons and matter. With no need for large-scale facilities, ED promises to take structure determination back to the laboratory. At the moment, it looks close to routine for chemical crystallography (CX) (Gruene *et al.*, 2018), but it has also been used to solve protein structures (Nannenga *et al.*, 2014; Shi *et al.*, 2013; Xu *et al.*, 2018). Recently, the first novel protein structure was solved by ED (Xu *et al.*, 2019). Besides simply solving protein structures, ED has the potential to go beyond MX. For one, the charged state of bound metals and protein side chains can be directly observed, although caution needs to be exercised in its interpretation (Yonekura *et al.*, 2015). In addition, hydrogen atoms scatter electrons relatively stronger than X-rays and are thus more easily visible in ED. Together, these advantages promise unprecedented information on the chemistry taking place during enzyme catalysis.

ED is nowhere near as established as X-ray crystallography. Electron diffractometers are not commercially available. As their design requirements are clear (Heidler *et al.*, 2019), they will probably be brought to the market fairly soon. Most algorithms for the processing of electron diffraction data and the refinement of the resulting models presume kinematic diffraction (i.e., each electron is diffracted only once, as in X-ray crystallography). Because of the strong interaction between electrons and matter, multiple (i.e., dynamical) diffraction is likely for all but the tiniest crystals. Data processing and model refinement thus result in poor statistics even if the data are good and models are accurate. The algorithms need to be adjusted to make comparison between X-ray and electron data possible and assess model quality with confidence (Palatinus *et al.*, 2015).

Other unresolved issues are radiation damage, which is rapid on tiny crystals but can be mitigated by a serial crystallography approach, and low data completeness caused by shadowing by sample supports developed for imaging. Data completeness can sometimes be improved by combining partial datasets from multiple crystals, but a preferred crystal orientation often makes this impossible. Nonflat sample supports have recently been introduced to help orient crystals randomly (Wennmacher *et al.*, 2019).

Once a suitable instrument with a fast and readout noise-free direct detector, a specifically developed sample stage with an accurate goniometer, a user-friendly control interface and software written for electron diffraction data exists, ED will see a dramatic rise in popularity. It will enable individual laboratories even at small institutions to set up microcrystallography facilities at moderate costs.

### Neutron diffraction

The only technique able to determine protein structures in the absence of radiation damage, at room temperature, and with hydrogen atoms clearly visible is neutron diffraction (Oksanen *et al.*, 2017). With it, the hydrogen bond networks surrounding active sites can be directly visualized, including the protonation states of contributing side chains (Gerlits *et al.*, 2019). Neutron diffraction should thus be much more popular than it is. However, few neutron sources for scientific use exist, data collection takes days, and sample requirements are highly restrictive. Crystals around 1 mm^3^ are often needed (Schröder *et al.*, 2018).

This disagreeable situation is slowly improving. Advances in detector technology and new experimental facilities will allow work on crystals of a size that was required for routine MX a decade ago. Nevertheless, it is difficult to see neutron diffraction becoming a mainstream technique within the next few years.

### Chemical crystallography

As a last possible specialization of MX beamlines, we would like to suggest chemical crystallography (CX). This has traditionally been done in the laboratory. Nearly every chemistry department has a crystallography facility where chemists can have the results of their syntheses verified. There are presently very few beamlines exclusively focused on CX (Barnett *et al.*, 2016). However, with the compounds under study getting bigger, the crystals tend to get smaller and diffract X-rays less well. Essentially, chemists are starting to experience some of the problems of MX. Adopting corresponding solutions is obvious (Clegg, 2019).

There are quite a few beamlines focusing on high-pressure and high-temperature crystallography and methods commonly summarized with the term extreme-condition crystallography (Itié *et al.*, 2015). These are somewhat outside the field of expertise of macromolecular crystallographers, but CX at ambient conditions is quite straightforward, despite the different vocabularies employed by the respective practitioners (Brink and Helliwell, 2019).

Beamline I19 at Diamond Light Source is set up to collect complete data with a minimum of complications (Allan *et al.*, 2017). The sample is rotated a few times with different crystal orientations. The detector remains in one position that covers reciprocal space from the lowest-resolution reflections right next to the direct beam to the highest-resolution reflections out at the opposite edge.

In contrast to the apparent simplicity of this particular setup, there are two competing requirements on data collection for CX that cannot easily be reconciled. Nonbiological materials frequently have a much higher heavy atom content than proteins or nucleic acids. Absorption and fluorescence can be serious problems. To minimize their effect, data collection at high energies is recommended, optimally with CdTe-based detectors. On the other hand, synthesized molecules, in particular in the pharmaceutical industry, need to have their absolute structure determined. This depends on anomalous signal much like experimental phasing in MX. If the compound does not contain heavy atoms, low energies must be used to maximize the anomalous signal.

In the future, we will see more specialized CX beamlines, CX beamlines for high-energy data collection, and MX beamlines modified to meet the needs of chemical crystallographers. With synchrotron beamlines keen to identify and attract loyal users, CX will play a much bigger role in the future than it does at the moment.

## CONCLUSION

MX has been incredibly successful at deciphering the atomic structures of biological macromolecules over the last forty-odd years when its supremacy has stood unchallenged. Thanks to technical developments, primarily in the area of detectors, cryo-EM has now emerged as a valid alternative. The two methods are complementary and should be treated as such. Neither is better or superior. They are in fact themselves complemented by the emerging method ED.

In the future, structural biology will be more integrative and look at the big picture. Structures of small proteins and isolated domains will continue to dominate the PDB, but their values will only be proven by putting them into a larger context, e.g., by fitting them into tomographic reconstructions of macromolecular complexes in their cellular context. We will understand biological connections at unprecedented detail.

To continue to play their important roles and contribute to the success of structural biology, synchrotron beamlines need to specialize. However, upgraded facilities, new equipment, and innovative methods are only one aspect of scientific success. The other, arguably more important, part is the questions they are helping to address. We envision that the structural biology community will use them and growing resources away from synchrotrons to confront the following fundamental open issues:

• What does structural biology teach us about biochemistry in cells? We need to critically assess structures obtained under cryoconditions and add the temperature domain to our experiments. This is critical for the understanding of processes taking place at physiological conditions.
• How can workflows for growing, identifying, and working with microcrystals be established and shared? They are necessary for ED, serial femtosecond crystallography, and serial crystallography at synchrotrons.
• How do transitions in biology and reactions in biochemistry occur over time? XFELs are good for the study of femto- to picosecond time scales. Upgraded synchrotrons will cover microsecond to second time scales. Cryo-EM is ideal for understanding the structural heterogeneity and conformational landscape of biological macromolecules in solution.
• What is the identity of chemically important ions in enzymes? Too much early work has been based on the best guesses.
• What are the electrostatic properties of side chains involved in interactions and chemistry? MX allows for guesses, but accurately determined cryo-EM and ED structures will yield interpretable data.
• What is the big picture? Getting a functional understanding of biology across several orders of magnitude requires integrative structural biology instead of dreams of superiority of individual methods. Time-resolved high-resolution structures of macromolecular complexes in their cellular context, with a range of functionally relevant binding partners and in a variety of conformational states determined at room temperature, require the combination of all methods at the structural biologist's disposal.

With a number of different techniques now capable of getting high-resolution information of biological macromolecules, structural biology has truly entered a golden age. We are excited about our understanding of biology in all its diversity growing dramatically over the next five years.
